# The Epilepsy of Infancy With Migrating Focal Seizures: Identification of *de novo* Mutations of the *KCNT2* Gene That Exert Inhibitory Effects on the Corresponding Heteromeric K_Na_1.1/K_Na_1.2 Potassium Channel

**DOI:** 10.3389/fncel.2020.00001

**Published:** 2020-01-24

**Authors:** Xiao Mao, Nadine Bruneau, Quwen Gao, Hélène Becq, Zhengjun Jia, Hui Xi, Li Shu, Hua Wang, Pierre Szepetowski, Laurent Aniksztejn

**Affiliations:** ^1^Hunan Provincial Maternal and Child Health Care Hospital, Changsha, China; ^2^NHC Key Laboratory of Birth Defects Research, Prevention and Treatment, Changsha, China; ^3^INSERM, Aix-Marseille University, INMED, UMR1249, Marseille, France; ^4^Department of Epilepsy, General Hospital of Southern Theater Command, Guangzhou, China

**Keywords:** epilepsy of infancy with migrating focal seizures, K_Na_ channels, *KCNT* genes, epilepsy, encephalopathy

## Abstract

The epilepsy of infancy with migrating focal seizures (EIMFS; previously called Malignant migrating partial seizures of infancy) are early-onset epileptic encephalopathies (EOEE) that associate multifocal ictal discharges and profound psychomotor retardation. EIMFS have a genetic origin and are mostly caused by *de novo* mutations in the *KCNT1* gene, and much more rarely in the *KCNT2* gene. *KCNT1* and *KCNT2* respectively encode the K_Na_1.1 (Slack) and K_Na_1.2 (Slick) subunits of the sodium-dependent voltage-gated potassium channel K_Na_. Functional analyses of the corresponding mutant homomeric channels *in vitro* suggested gain-of-function effects. Here, we report two novel, *de novo* truncating mutations of *KCNT2*: one mutation is frameshift (p.L48Qfs43), is situated in the N-terminal domain, and was found in a patient with EOEE (possibly EIMFS); the other mutation is nonsense (p.K564*), is located in the C-terminal region, and was found in a typical EIMFS patient. Using whole-cell patch-clamp recordings, we have analyzed the functional consequences of those two novel *KCNT2* mutations on reconstituted K_Na_1.2 homomeric and K_Na_1.1/K_Na_1.2 heteromeric channels in transfected chinese hamster ovary (CHO) cells. We report that both mutations significantly impacted on K_Na_ function; notably, they decreased the global current density of heteromeric channels by ~25% (p.K564*) and ~55% (p.L48Qfs43). Overall our data emphasize the involvement of *KCNT2* in EOEE and provide novel insights into the role of heteromeric K_Na_ channel in the severe *KCNT2*-related epileptic phenotypes. This may have important implications regarding the elaboration of future treatment.

## Introduction

**Figure 1:**
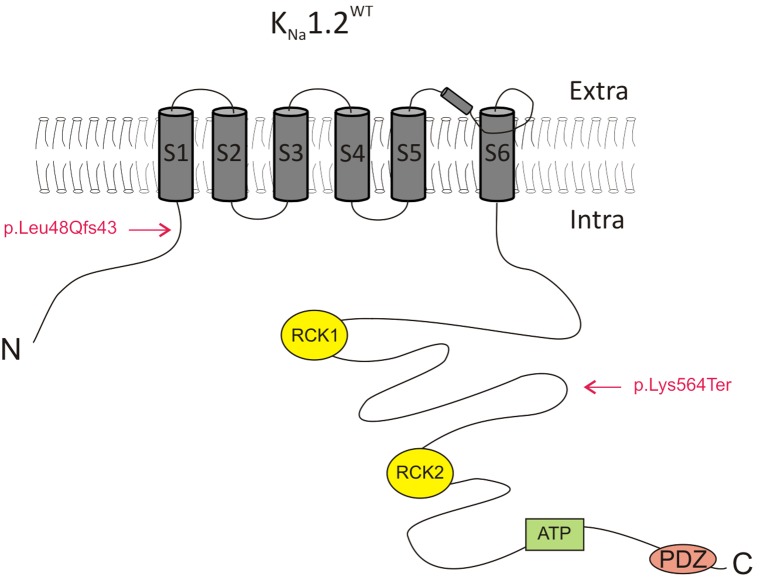
Schematic representation of the K_Na_1.2 subunit. The location of the two mutations identified in the present study (p.K564* and p.L48Qfs43) is indicated. ATP, adenosine triphosphate binding domain; Extra, extracellular milieu; Intra, intracellular milieu. RCK1 and RCK2, regulator of conductance of K^+^ domains 1 and 2.

Channelopathies represent an important cause of neurological disorders (Kumar et al., 2016). Dysfunction of potassium channels has notably been involved in various types of epileptic encephalopathies, including epilepsy of infancy with migrating focal seizures (EIMFS), previously known as malignant migrating partial seizures of infancy. EIMFS are rare, neonatal epilepsies characterized by onset before the age of 6 months, and usually during the first weeks of life, by continuous migrating polymorphous focal seizures with corresponding multifocal ictal electroencephalographic (EEG) discharges associated with progressive deterioration of psychomotor development (Coppola et al., 1995). EIMFS have a genetic origin and can be caused by *de novo* mutations in the *KCNT1* gene encoding the K_Na_1.1 subunit (Slack or Slo2.2) of K_Na_ channels (Barcia et al., 2012; Ishii et al., 2013; McTague et al., 2013; Rizzo et al., 2016). More recently, two pathogenic mutations in the *KCNT2* gene encoding the K_Na_1.2 subunit (Slick or Slo2.1) have been reported (Gururaj et al., 2017; Ambrosino et al., 2018). K_Na_ channels are voltage-gated potassium channels that are activated by an increase of cytoplasmic Na^+^ concentration. They contribute to the slow afterhyperpolarization that follows a train of the action potential in several neuronal populations of the brain (Stafstrom et al., 1985; Kim and McCormick, 1998; Budelli et al., 2009; Hage and Salkoff, 2012; Kaczmarek, 2013; Kaczmarek et al., 2016). These subunits co-assemble to form homo or tetra-heteromeric K_Na_ channels. Each subunit is composed of six transmembrane segments and of two intracellular N and C terminal domains (Figure 1). These two subunits display structural differences notably regarding their distal C-terminal region, their electrophysiological properties, their responses to neuromodulators, and their sensitivities to changes in cell volume (Bhattacharjee et al., 2003; Santi et al., 2006; Kaczmarek, 2013; Tejada et al., 2017). The C terminal part contains consensus sites for Na^+^ within the RCK2 (regulator of conductance of K^+^) domain and interaction sites for cytoplasmic proteins (e.g., protein kinase C). In K_Na_1.2 but not K_Na_1.1, the C-terminus also harbors a binding site for ATP, which function remains elusive (Bhattacharjee et al., 2003; Berg et al., 2007; Kaczmarek, 2013; Garg and Sanguinetti, 2014; Kaczmarek et al., 2016; Gururaj et al., 2017). In heterologous cells, functional analysis of mutant channels associated with EIMFS mostly revealed gain of function effects: potassium current was increased in cells expressing homomeric K_Na_1.1 channels harboring either of the p.Val271Phe, p.Gly288Ser, p.Arg398Gln, p.Arg428Gln, p.Arg474His, p.Met516Val, p.Lys629Asn, p.Ile760Met, p.Pro924Leu or p.Ala934Thr missense mutations, and in cells expressing homomeric K_Na_1.2 channels harboring either of the p.Arg190His or p.Arg190Pro missense mutations (Barcia et al., 2012; Rizzo et al., 2016; Villa and Combi, 2016; Ambrosino et al., 2018). A change in channel function has also been described in cells expressing the K_Na_1.2 subunit harboring the p.Phe240Leu missense mutation: the mutant channel lost its selectivity to K^+^ ions and gained permissiveness to Na^+^ ions (Gururaj et al., 2017).

Here, we have used exome sequencing to identify two novel *de novo* nonsense and frameshift mutations of the *KCNT2* gene in two patients with ascertained EIMFS and with EIMFS-like early-onset epileptic encephalopathies (EOEE), respectively. We have investigated the functional consequences of the two mutations in heterologous cells expressing heteromeric channels and showed that both mutations reduced whole-cell potassium current. Therefore, EIMFS may be caused not only by an increase but also by a decrease in the function of K_Na_.

## Materials and Methods

### Patients

The two patients with *KCNT2* mutations were recruited at Hunan Provincial Maternal and Child Health Care Hospital. Leukocyte DNA was extracted from peripheral blood stored in EDTA tubes by the phenol-chloroform method. Clinical information was collected by experienced neurologists. Patients’ parents had informed consents and the study was approved by the Ethics Committee of Hunan Provincial Maternal and Child Health Care Hospital.

### Exome Sequencing

Patients’ DNAs were analyzed by next-generation sequencing with the whole-exome sequencing (WES) approach. DNA fragments were sequenced on the HiSeq2500 system (Illumina, San Diego, CA, USA) with a mean depth of 100×. A preliminary processing of WES data (data alignment and filter) followed pipelines as previously reported (Wang et al., 2011).

The annotated data by ANNOVAR (version 20160201; Wang et al., 2011) was used for further data analyses. Public databases (1000Genome, ESP6500, ExAC, dbSNP, and gnomAD) were used to filter known variants with minor allele frequencies (MAFs) over 0.001. Bioinformatics software (PolyPhen, SIFT, CADD or Mutation Taster) were used to predict the pathogenicity of single-nucleotide variations (SNVs). The loss-of-function variants (nonsense variants, frameshift variants, and splicing variants) and predicted pathogenic SNVs were retained. ACMG guidelines were finally used to evaluate the pathogenicity of the variants (Richards et al., 2015).

Sanger sequencing was performed on patients’ DNA to validate the findings of WES and on parents’ DNA to study familial inheritance. Sequencing primers were designed according to the sequences of the detected variants and polymerase chain reaction amplification was carried out for Sanger sequencing.

### Constructs and Site-Directed Mutagenesis

The human *KCNT1* cDNA construct (Genecopoeia EX-Y5001-M02, thereafter designated as pKCNT1) was used for the expression of wild-type human K_Na_1.1 subunit (NM_020822). Two human *KCNT2* cDNA constructs (Genecopoeia EX-Y5628-M61and EX-Y5628-M83, thereafter designated as pKCNT2-ires-GFP and pKCNT2-ires-mCherry, respectively) were used for expression of mutant or wild-type human K_Na_1.2 subunits (NM_198503) coupled with internal ribosome entry site (IRES)-driven independent expression of eGFP (green fluorescent protein) or mcherry protein, respectively.

Mutant *KCNT2* constructs were generated from their wild-type counterpart by using QuikChange Lightning Site-Directed Mutagenesis Kit according to the manufacturer’s protocol (Agilent Technologies) and the following forward and reverse primers: *KCNT2-A1690T*: 5′-tctctgctggtcttggttttaaaatgctgaattctcttctttg and 5′-caaagaagagaattcagcattttaaaaccaagaccagcagaga (K_Na_1.2^K564*^)*; KCNT2-del143-144*: 5′-cttgatctctggttttttatgaaaaataatttgtctttctttaaatgtattttcattcatatag and 5′-ctatatgaatgaaaatacatttaaagaaagacaaattatttttcataaaaaaccagagatcaag(K_Na_1.2^L48Qfs43^). *KCNT2* sequences from wild-type and mutant constructs were all verified by Sanger sequencing (GATC Biotech).

### Cell Cultures and Transfections

Chinese hamster ovary (CHO) cells were cultured at 37°C in a humidified atmosphere with 5% CO_2_ with an F-12 Nutrient Mixture (Life Technologies) supplemented with 10% FBS (Fetal Bovine Serum) and 100 units/ml antibiotics/antimycotics (Life Technologies). These cells were transiently transfected using the Neon^®^ Transfection System (Life Technologies) according to the manufacturer’s protocol. Briefly, 10^5^ cells in suspension were transfected with a total amount of 2 μg of DNA. Non-recombinant pcDNA3.1 was added if necessary and concentrations were adjusted to get a total amount of 2 μg of DNA. Electroporation configuration was 1,400 V, 1 pulse, 20 ms. Cells were transiently co-transfected with pKCNT1 and either of pKCNT2-Mutant-ires-GFP or pKCNT2-WT-ires-mCherry or both (see below). Following electroporation, cells were cultured on pre-coated glass coverslips and maintained at 37°C and 5% CO_2_ with a complete medium for 2 days before recordings. Combinations of plasmids used in this study are shown in Table 1. The eGFP and mCherry fluorescent dyes were used to ascertain the efficacy of transfection assays and select cells for recordings.

**Table 1:** Biophysical properties of currents recorded in chinese hamster ovary (CHO) cells transfected with the following plasmid combinations.

	Transfected plasmid (μg)	*n*	Current density @+60 mV	V_1/2_ (mV)	k (mV/efold)
*peGFP + pcDNA3.1*	1 + 1	9	30.8 ± 4.2		
**Homozygous configurations**					
*KCNT2-ires-GFP + pcDNA3.1* (K_Na_1.2^WT^)	1 + 1	12	46.7 ± 5.6 (without ATP)		
*KCNT2-ires-GFP + pcDNA3.1* (K_Na_1.2^WT^)	1 + 1	6	50.8 ± 12.1 (with ATP)		
*KCNT2-A1690T-ires-GFP + pcDNA3.1* (K_Na_1.2^K564*^)	1 + 1	10	31.9 ± 4.4		
*KCNT2-del143-144-ires-GFP + pcDNA3.1* (K_Na_1.2^L48Qfs43^)	1 + 1	10	24.6 ± 3.9		
*KCNT2-ires-mcherry + KCNT2-A1690T-ires-GFP* (K_Na_1.2^WT^ + K_Na_1.2^K564*^)	1 + 1	11	34.5 ± 6.9		
*KCNT2-ires-mcherry + KCNT2-del143-144-ires-GFP* (K_Na_1.2^WT^ + K_Na_1.2^L48Qfs43^)	1 + 1	10	26.0 ± 4.2		
*KCNT1 + pcDNA3.1 + peGFP* (K_Na_1.1)	1 + 0.5 + 0.5	25	371.8 ± 39.7	10.7 ± 3.3	42.9 ± 3.3
**Heterozygous configurations**					
*KCNT1 + KCNT2-ires-GFP* (K_Na_1.1 + K_Na_1.2^WT^)	1 + 1	21	629.4 ± 63.5	−1.3 ± 5.1	44.6 ± 5.2
*KCNT1 + KCNT2-A1690T-ires-GFP* (K_Na_1.1^WT^ + K_Na_1.2^K564*^)	1 + 1	18	405.8 ± 46.0	6.8 ± 4.2	33.8 ± 3.1
*KCNT1 + KCNT2-del143-144-ires-GFP* (K_Na_1.1^WT^ + K_Na_1.2^L48Qfs43^)	1 + 1	19	369.8 ± 51.1	−6.5 ± 4.3	42.7 ± 3.7
*KCNT1 + KCNT2-ires-mcherry + KCNT2-A1690T-ires-GFP* (K_Na_1.1 + K_Na_1.2^WT^ + K_Na_1.2^K564*^)	1 + 0.5 + 0.5	18	496.3 ± 54.7	4.7 ± 5.61	41.0 ± 3.5
*KCNT1 + KCNT2-ires-mcherry + KCNT2-del143-144-ires-GFP* (K_Na_1.1 + K_Na_1.2^WT^ + K_Na_1.2^L48Qfs43^)	1 + 0.5 + 0.5	14	332.8 ± 39.8	7.9 ± 4.3	33.1 ± 3.3

*n, number of recorded cells. Values of voltage for half-maximal activation of potassium channel (V_1/2_) and of slope factor (k) of conductance/voltage relationships are indicated*.

### Electrophysiology

CHO cells were perfused at 1–2 ml/min with the following solution (in mM): 135 NaCl, 3.5 KCl, 5 NaHCO_3_, 0.5 NaH_2_PO_4_, 1 MgCl_2_, 1.5 CaCl_2_, 10 HEPES, 10 glucose, and pH 7.3 adjusted with NaOH. Whole-cell patch-clamp recordings were performed with microelectrodes (borosilicate glass capillaries GC 150F-15, Harvard apparatus) filled with a solution containing (in mM): 135 KCl, 0.1 CaCl_2_, 1.1 EGTA, 10 HEPES, 3 Mg^2+^ATP, 0.3 Na^+^GTP, 4 phosphocreatine, pH 7.3 adjusted with KOH and a resistance of 4–6 MΩ. In some experiments, ATP and GTP were omitted from the internal pipette solution. Data were sampled at 10 kHz and filtered with a cut-off frequency of 3 kHz using an EPC-10 amplifier (HEKA Electronik). An hyperpolarizing voltage step of 10 mV during 500 ms followed by incremental depolarizing voltage steps command of 10 mV was applied from a holding potential of −90 mV and up to +110 mV in order to analyze current densities and the conductance–voltage (G–V) relationships. Current densities (expressed in pA/pF) were calculated by measuring current amplitude at the end of the voltage step divided by the capacitance (Cm). G values were obtained from peak amplitudes of the slow outward current divided by the driving force for K^+^ ions with E_K_ ~−93 mV and normalized to the maximal conductance. Plotted points were fitted with a Boltzmann function: G/Gmax = 1/[1 + exp(V1/2 − Vm)/k] to yield the voltage for half-maximum activation (V_half_) and the slope factor (k) values. Currents were analyzed using Origin 8.0 software. Analyses were performed after offline leak current subtraction. Membrane potentials were corrected for liquid junction potential (~5 mV).

### Statistical Analyses

Data are represented as means ± SEM. Two-way ANOVA with Tukey’s correction for multiple testing or Kruskal–Wallis test were used to assess statistical significance; *adjusted *p* < 0.05; **adjusted *p* < 0.01; ***adjusted *p* < 0.001.

## Results

### Identification of Two Novel *de novo* KCNT2 Defects in Patients With Early-Onset Epileptic Encephalopathies

**Figure 2:**
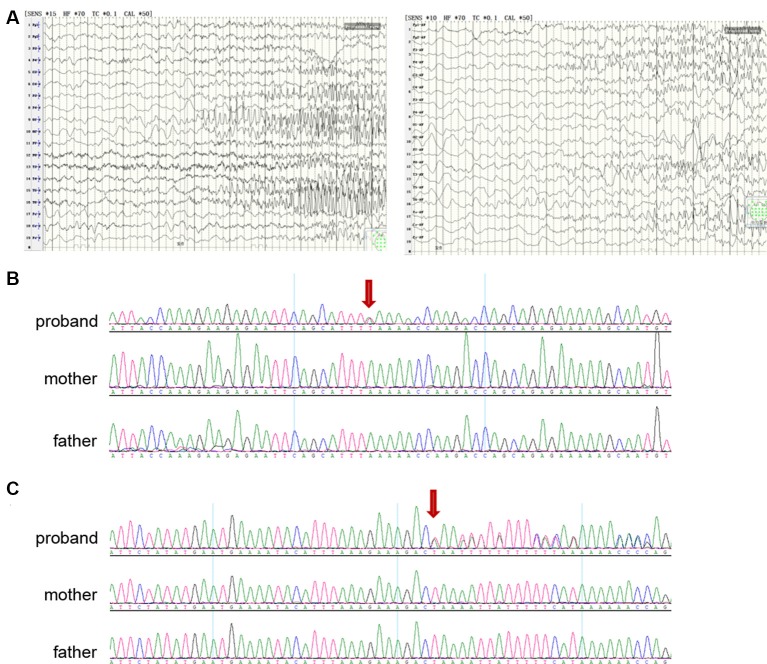
Clinical and genetic data. **(A)** Electroencephalographic (EEG) of a proband with epilepsy of infancy with migrating focal seizures (EIMFS; patient A), showing seizures arising from different hemispheres (left: left occipital lobe; right: right frontal lobe). **(B)** Sanger sequencing showing (red arrow) nonsense variant p.K564* (NM_198503.2:c.1690A>T) in *KCNT2* in the proband (patient A) and not in the parents. **(C)** Sanger sequencing showing (red arrow) frameshift variant p.L48Qfs43 (NM_198503.2:c.143-144 delTA) in *KCNT2* in the proband (patient B) and not in the parents.

Patient A, female, was born at 42 weeks of gestation after normal pregnancy and delivery. She is the first child of healthy non-consanguineous parents. At 2 months of age, she started to have seizures characterized by twitches of the eyelids, tonic elevation of a single limb or both limbs, and perioral cyanosis. The seizures usually lasted several minutes and occurred in clusters with an increasing frequency of more than 20 seizures per day at 3 months. Neurologic examination revealed generalized hypotonia and severe neurologic impairment with the poor visual following. Seizures were refractory to various antiepileptic drugs including valproate, lamotrigine and levetiracetam. Brain magnetic resonance imaging (MRI) was normal. EEG showed a symmetric slow background pattern, multifocal spikes and seizures arising from different regions independently and migrating from one hemisphere to the other at times (Figure 2A).

DNA from patient A were screened by WES and analyzed by Clinical Sequencing Analyzer (CSA of WuXiNextCODE). After applying filtering methods, Sanger sequencing was performed to exclude false-positive and examine inheritance. We identified a *de novo* nonsense variant p.K564* (NM_198503.2:c.1690A>T; ClinVar accession number: VCV000695093.1) in *KCNT2* which was absent from controls in ExAC, gnomAD, 1000 Genomes, ESP6500 and dbSNP databases, and compound heterozygous variants in *ABCC2* (NM_000392.3:c.1018C>A and c.1313T>G). No other variant of interest was identified in other genes including known epilepsy genes. Pathogenic variants in *ABCC2* can cause Dubin-Johnson syndrome, a benign autosomal recessive disorder characterized by hyperbilirubinemia with no clinical feature shared with our patients. In the course of the present study, Gururaj et al. (2017) and Ambrosino et al. (2018) identified two *de novo* *KCNT2* missense variants in patients with epileptic encephalopathy. Despite the fact that the *KCNT2* gene would not be highly intolerant to loss-of-function mutations, as a few nonsense variants have been detected in control individuals and the pLI score (the probability of being loss of function intolerant) is at 0.67 only (see the ExAC database at: http://exac.broadinstitute.org/), we considered the *de novo* nonsense variant p.K564* as the most plausible genetic cause (Figure 2B).

We then searched additional *KCNT2* variants in our in-house WES database of a cohort of more than 200 patients with early-onset epileptic encephalopathy (EOEE). We found a *de novo* frameshift variant p.L48Qfs43 (NM_198503.2:c.143-144 delTA; ClinVar accession number: VCV000695094.1) in *KCNT2* in an EOEE patient (Patient B) and validated this variant by Sanger sequencing (Figure 2C). This frameshift variant was absent from controls in control databases and no other variant of interest was found in known causative epilepsy genes including EIMFS. Patient B is a 29 years old female who showed mild intellectual disability and seizures. According to her parents and to the medical records, the patient began to have seizure attacks when she was 4 months old. Seizures were mainly focal and migrating, which likely corresponded to EIMFS. However, due to the fact that this is an aged case and considering the relatively low medical level in China almost 30 years ago, diagnosis cannot be firmly ascertained.

### Functional Analysis of Wild Type and Mutant Homomeric Human K_Na_1.2 Channels

**Figure 3:**
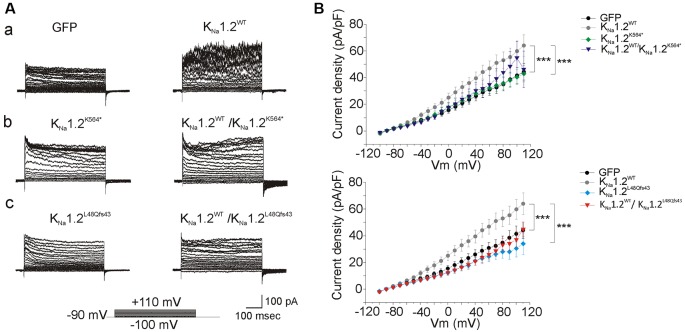
Functional analysis of homomeric wild type and mutant human K_Na_1.2 channels in chinese hamster ovary (CHO) cells. **(A)** Current response to 10 milliVolts (mV) voltage steps command from −90 mV to +110 mV for 500 ms in cells transfected with plasmids encoding: (a) green fluorescent protein (GFP, left traces), wild-type K_Na_1.2 (K_Na_1.2^WT^, right traces) subunits; (b) the mutant p.K564* (K_Na_1.2^K564*^, left traces), both K_Na_1.2^WT^ and K_Na_1.2^K564*^ subunits (right traces); (c) the mutant p.L48Qfs43 (K_Na_1.2^L48Qfs43^, left traces), both K_Na_1.2^WT^ and K_Na_1.2^L48Qfs43^ subunits (right traces). **(B)** Upper graph: current density expressed in picoAmperes/picofarads (pA/pF) measured at membrane potentials (Vm) as indicated in abscissa, in cells transfected with plasmids encoding GFP, K_Na_1.2^WT^, K_Na_1.2^K564*^, both K_Na_1.2^WT^, and K_Na_1.2^K564*^ subunits. Bottom graph: same measurements in cells transfected with plasmids encoding GFP, K_Na_1.2^WT^, K_Na_1.2^L48Qfs43^, both K_Na_1.2^WT^ and K_Na_1.2^L48Qfs43^ subunits. Corresponding symbols are shown on the right of each graph. Two-way ANOVA with Tukey’s correction for multiple comparisons. ***p < 0.001.

To investigate the functional consequences of the K_Na_1.2 (*KCNT2*) mutations, CHO cells were first transfected with plasmids encoding the wild type K_Na_1.2 (K_Na_1.2^WT^) subunit. Two days later, cells were recorded with a KCl filled pipette solution that did not contain ATP, as this nucleotide which binds the C-terminal domain of K_Na_1.2 inhibits channel activity (Bhattacharjee et al., 2003; but see Berg et al., 2007; Garg and Sanguinetti, 2014; Gururaj et al., 2017). We observed that whole-cell current density was slightly but significantly higher than in cells transfected with control plasmid encoding GFP only (*n* = 12 and 9 cells respectively, Figure 3; Table 1). Values were close to those reported recently in HEK cells (Ambrosino et al., 2018). Similar recordings with pipette solution containing ATP yielded the same results (*n* = 6 cells, Table 1). This suggested that low expression of K_Na_1.2 channel-mediated current in CHO cells is independent of the presence or absence of ATP.

CHO cells were then transfected with plasmids encoding either the human K_Na_1.2 p.K564* (K_Na_1.2^K564*^; *n* = 10 cells) or the K_Na_1.2 p.L48Qfs43 (K_Na_1.2^L48Qfs43^; *n* = 10 cells) mutant subunits. p.K564* is a nonsense mutation localized in the C-terminal part of K_Na_1.2 and situated between the RCK1 and RCK2 domains (Figure 1). This mutation leads to a truncated protein lacking the RCK2 domain and the ATP and PDZ binding sites. p.L48Qfs43 is a frameshift mutation localized in the N-terminal domain of the protein. This mutation leads to a protein composed of the N-terminal domain and the first transmembrane segment S1. For both mutations, depolarizing voltage steps elicited significantly smaller currents compared to cells transfected with K_Na_1.2^WT^ plasmids (*n* = 12 cells), and responses were similar to those obtained in cells expressing only GFP. Same results were obtained in cells co-transfected with plasmids encoding K_Na_1.2^WT^ and K_Na_1.2^K564*^ subunits (*n* = 11 cells), or K_Na_1.2^WT^ and K_Na_1.2^L48Qfs43^ subunits (*n* = 10 cells, Figure 3). Although the current mediated by K_Na_1.2^WT^ was very small, these data suggested that in contrast with other previously reported *KCNT2* mutations (Ambrosino et al., 2018), p.K564* and p.L48Qfs43 decreased K_Na_1.2-mediated currents.

### Functional Analysis of Wild Type and Mutants Heteromeric K_Na_1.1/K_Na_1.2 Channels

Immunohistochemical studies performed in rodent brain have shown that K_Na_1.1 and K_Na_1.2 subunits exhibited distinct expression patterns but could also co-localize (Bhattacharjee et al., 2005; Chen et al., 2009; Rizzi et al., 2016). Moreover, biochemical and electrophysiological studies performed in heterologous cells have demonstrated that rat K_Na_1.1 and rat K_Na_1.2 subunits can form heteromeric channels (Chen et al., 2009). The co-assembly of the two subunits enhances channel expression to the plasma membrane, leading to the global current density that is higher than with K_Na_1.1 or K_Na_1.2 alone (Chen et al., 2009). We thus decided to study if the same properties would also characterize the human K_Na_ subunits, and if so, to analyze the impact of the two pathogenic mutations in this heteromeric condition.

**Figure 4:**
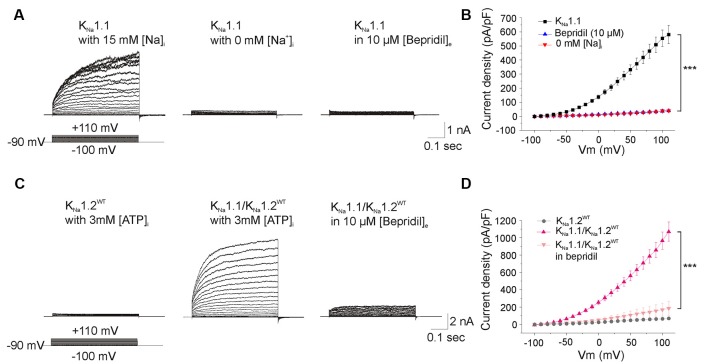
Functional analysis of K_Na_1.1 and heteromeric K_Na_1.1/K_Na_1.2 channels. **(A)** Current traces evoked by voltage steps in cells expressing K_Na_1.1 subunit and recorded with pipette solution containing either 15 mM Na^+^ (left traces), or no Na^+^ (middle traces), or 15 mM Na^+^ and an extracellular medium containing 10 μM bepridil (right traces). **(B)** Current densities measured in the three recording conditions as in **(A)**. **(C)** Current traces recorded with pipette solution containing 3 mM ATP/0.3 mM GTP in cells expressing the K_Na_1.2^WT^ subunit (left traces), both K_Na_1.1 and K_Na_1.2^WT^ subunits (middle traces) and both K_Na_1.1 and K_Na_1.2^WT^ subunits in the presence of 10 μM bepridil in the extracellular medium (right traces). **(D)** Current densities measured in the three conditions as depicted in **(C)**. Two-way ANOVA with Tukey’s correction for multiple comparisons. ***p < 0.001.

To this aim, CHO cells were first transfected with plasmid encoding K_Na_1.1 and recorded with a KCl filled pipette solution containing ATP. These cells responded to depolarizing voltage steps by large outwardly rectifying currents (*n* = 25 cells, Figures 4A,B). Currents were abolished in cells superfused with bepridil 10 μM (*n* = 5 cells), or in cells recorded with a Na^+^-free internal pipette solution (*n* = 5 cells, Figures 4A,B). These data confirmed that the outward rectifying current was mediated by activation of the Na^+^-dependent potassium K_Na_ channels. Boltzmann analysis of the conductance/voltage curve showed that V_half_ was at 10.7 ± 3.3 mV, a mean value close to the one reported previously (Rizzo et al., 2016), and the slope factor was at 42.9 ± 3.3 mV/e fold (*n* = 17 cells, Table 1), a mean value higher than the one reported by the same authors but which indicated the low voltage sensitivity of K_Na_ channels (Salkoff et al., 2006).

**Figure 5:**
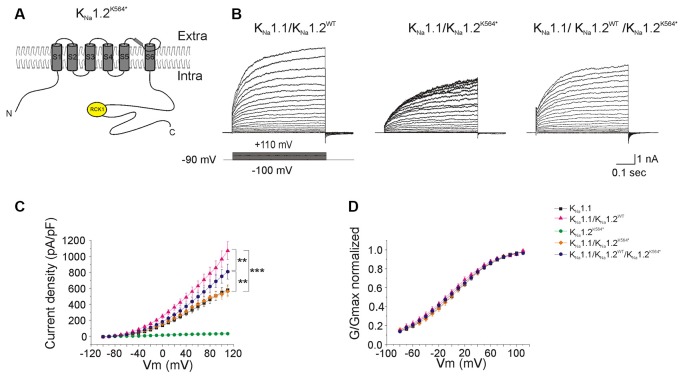
Functional consequences of the p.K564* mutation on heteromeric channels. **(A)** Schematic representation of the expected K_Na_1.2^K564*^ mutant subunit. The nonsense mutation locates in the C-terminal part of the protein between the RCK1 and RCK2 domains, leading to a predicted truncated protein. **(B)** Representative current responses to depolarizing voltage steps in CHO cells transfected with K_Na_1.1 + K_Na_1.2^WT^ (left traces); K_Na_1.1 + K_Na_1.2^K564*^ (middle traces); K_Na_1.1 + K_Na_1.2^WT^ + K_Na_1.2^K564*^ (right traces) plasmids. **(C)** Current densities measured in these three conditions but also including K_Na_1.1 and K_Na_1.2^WT^ for comparison. **(D)** Conductance-voltage relationship of wild type homomeric K_Na_1.1; wild type heteromeric K_Na_1.1 + K_Na_1.2; heteromeric mutant K_Na_1.1 + K_Na_1.2^K564*^ and heteromeric mutant K_Na_1.1 + K_Na_1.2^WT^+ K_Na_1.2^K564*^ channels normalized. Two-way ANOVA with Tukey’s correction for multiple comparison. ***p < 0.001; **p < 0.01.

CHO cells were then transfected with plasmids encoding the K_Na_1.1 and K_Na_1.2^WT^ subunits (*n* = 21 cells). We observed that whole-cell current was almost twice higher than that generated by K_Na_1.1 alone (*n* = 25 cells, Figures 4C,D), without any significant change in V_half_ and in the slope factor of the conductance/voltage relationship (Figure 5, Table 1). This current was also dramatically reduced by bepridil 10 μM (*n* = 5 cells). Thus, like for rat K_Na_1.1 and rat K_Na_1.2, the two human subunits might co-assemble in CHO cells to form heteromeric channels with larger currents.

We then evaluated the consequences of each of the two mutant K_Na_1.2 subunits on heteromeric K_Na_1.1/K_Na_1.2 channels, either in a homozygous state, or in a heterozygous state to mimic the patient’s situation. In cells co-transfected with K_Na_1.1 and K_Na_1.2^K564*^ plasmids (homozygous mutant state, *n* = 18 cells), the level of whole-cell current was lower as compared to the wild-type situation, and was similar to the current recorded in cells expressing K_Na_1.1 only (Figures 5A,B). The reduction of global current density was not associated with any significant change in the conductance-voltage relationship (*n* = 15 cells). In cells co-transfected with K_Na_1.1/K_Na_1.2^WT^/K_Na_1.2^K564*^ plasmids (heterozygous mutant state, *n* = 18 cells), the level of whole-cell current was significantly increased as compared with cells expressing K_Na_1.1 channels only and was significantly decreased as compared with cells expressing heteromeric wild-type K_Na_1.1/K_Na_1.2^WT^ channels (Figures 5B–D, Table 1).

**Figure 6:**
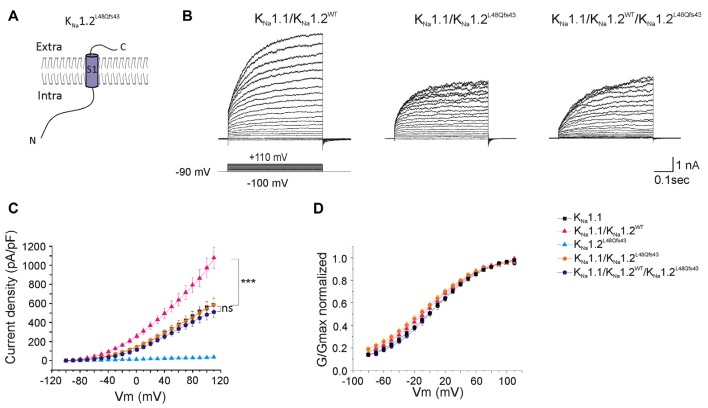
Functional consequences of the p.L48Qfs43 mutation on heteromeric channels. **(A)** Schematic representation of the expected K_Na_1.2^L48Qfs43^ mutant subunit. The frameshift mutation locates in the N-terminal part of the protein, leading to predicted protein composed of the N-terminal domain and of transmembrane segment S1. **(B)** Representative current responses to depolarizing voltage steps in CHO cells transfected with K_Na_1.1 + K_Na_1.2^WT^ (left traces); K_Na_1.1 + K_Na_1.2^L48Qfs43^ (middle traces); K_Na_1.1 + K_Na_1.2^WT^+ K_Na_1.1^L48Qfs43^ (right traces) plasmids. **(C)** Current densities measured in these three conditions but also including K_Na_1.1 and K_Na_1.2^WT^ for comparison. Corresponding symbols are indicated on the top of the graph. **(D)** Conductance-voltage relationship of wild type homomeric K_Na_1.1; wild type heteromeric K_Na_1.1 + K_Na_1.2; heteromeric mutant K_Na_1.1 + K_Na_1.2^L48Qfs43^ and heteromeric mutant K_Na_1.1 + K_Na_1.2^WT^+ K_Na_1.2^L48Qfs43^ channels normalized. Two-way ANOVA with Tukey’s correction for multiple comparisons. ***p < 0.001; ns, not significant.

As with the p.K564* mutation, whole-cell current measured in cells co-transfected with K_Na_1.1 and mutant K_Na_1.2^L48Qfs43^ plasmids (homozygous mutant state, *n* = 19 cells, Figures 6A,B) was identical to the current measured in cells transfected with K_Na_1.1 plasmid only (*n* = 25 cells), again without any significant change in the conductance/voltage relationship. Interestingly, currents measured in cells co-transfected with K_Na_1.1/K_Na_1.2^WT^/K_Na_1.2^L48Qfs43^ plasmids (*n* = 14 cells) were identical to currents measured in cells expressing K_Na_1.1 only (Figures 6B–D, Table 1). This indicated a possible dominant-negative effect for p.L48Qfs43.

## Discussion

Here, we report two patients diagnosed as EIMFS and EIMFS-like EOEE, respectively, and carrying two novel *de novo* variants in the *KCNT2* gene. Patient A in our study fulfilled the diagnostic criterion for EIMFS (Coppola et al., 1995) including age at the onset before 6 months of age, migrating focal motor seizures, seizures refractory to antiepileptic drugs, and severe psychomotor delay. EEG of patient A also showed the typical “jumping” areas at onset between two hemispheres. According to the clinical manifestations and the EEGs, diagnosis of EIMFS in patient A can be ascertained. We also diagnosed patient B as probably having EIMFS, based on the age at onset and characteristics of her seizure attacks.

EIMFS is a severe, drug-resistant, early-onset epilepsy encephalopathy in which variants in the *KCNT1*, *SCN1A*, *SCN8A*, *SCN2A*, *PLCB1*, *KCNT1*, *SLC25A22*, *TBC1D24* and *SLC12A5* genes as well as 16p11.2 duplication have been reported. While *de novo* gain of function *KCNT1* (K_Na_1.1) variants are the most common cause of EIMFS (Bedoyan et al., 2010; Carranza Rojo et al., 2011; Barcia et al., 2012; Poduri et al., 2012, 2013; Ishii et al., 2013; McTague et al., 2013; Milh et al., 2013; Ohba et al., 2014; Howell et al., 2015; Stödberg et al., 2015; Rizzo et al., 2016; Villa and Combi, 2016), it was recently reported that *de novo* mutations in the *KCNT2* (K_Na_1.2) gene also caused EOEE, including EIMFS. Two gain-of-function *KCNT2* mutations were identified in a patient with West syndrome that evolved to a Lennox-Gastaut syndrome, and in a patient with EIMFS, respectively (Ambrosino et al., 2018). A third *KCNT2* mutation leading to K_Na_1.2 channels that were no more selective for K^+^ ions and that became permeable to Na^+^ ions was identified in a patient with multi-focal epileptogenic activity or hypsarrhythmia (Gururaj et al., 2017). Here, we have identified in two patients with EIMFS and EIMFS-like phenotypes, two novel *de novo* mutations in *KCNT2* (K_Na_1.2), respectively localized in the N-terminal (p.L48Qfs43) and C-terminal (p.K564*) domains of the protein and which both led to significantly reduced activity of heteromeric K_Na_ channels *in vitro*. To reach this conclusion, we have analyzed the macroscopic current in CHO cells co-transfected with wild type and mutant K_Na_1.2 subunits, notably in the heterozygous configuration to mimic the patient’s situation. To the best of our knowledge, the functional impact of K_Na_1.2 pathogenic mutations on currents generated by human heteromeric K_Na_ channels had never been tested in such a configuration, although immunohistochemical studies performed in adult rodent brain provided evidence that K_Na_1.1 and K_Na_1.2 subunits can co-localize and potentially form heteromeric channels (Bhattacharjee et al., 2005; Chen et al., 2009; Rizzi et al., 2016). K_Na_ channels are also very likely to be formed by homomeric K_Na_1.1 or K_Na_1.2 subunits in another large subset of neuronal cells. Here, we observed that human K_Na_1.2^WT^ produced a very small current either in the presence or absence of ATP. The same difficulty to detect current in CHO cells was mentioned by Gururaj et al. (2017). In cells co-transfected with K_Na_1.1 and K_Na_1.2 plasmids, the current was larger as compared to cells transfected either with K_Na_1.1 or with K_Na_1.2 plasmids alone, indicating that both subunits are expressed and would co-assemble to form a heteromeric channel. It is possible that in spite of the internal dialysis due to whole-cell recording, the endogenous concentration of ATP in CHO cells is high enough to exert its inhibitory effect on the K_Na_1.2 subunit (Bhattacharjee et al., 2003)—although the action of ATP has been challenged in other studies (Berg et al., 2007; Garg and Sanguinetti, 2014; Gururaj et al., 2017). Another possibility is that the intracellular concentration of Na^+^ (15 mM) is not high enough to activate the channel. The reliable current was observed in HEK cells expressing human K_Na_1.2 subunit but with a patch pipette solution containing 70 mM Na^+^ concentration (Berg et al., 2007). In fact, K_Na_1.2 channels might be less sensitive to [Na^+^]_i_ than K_Na_1.1 (Bhattacharjee et al., 2003; Kaczmarek, 2013). The small current produced by human K_Na_1.2 contrasts with the large whole-cell current produced in the same cells by rat K_Na_1.2 subunit (Gururaj et al., 2017). There are slight differences in amino acids sequence between the rat and human K_Na_1.2 subunits (~2%; Bhattacharjee et al., 2003): some of these amino acids may be instrumental for the functional discrepancy between rat and human K_Na_1.2 subunit, as shown for the rodent and human Kv7.3 subunit of Kv7 potassium channels (Etxeberria et al., 2004). Thus, the role of homomeric K_Na_1.2 channel in human neurons might even be questioned if, like in CHO cells, they mediated a very small current only. Hence and apart from the gain of function mutations reported recently (Ambrosino et al., 2018), the role of K_Na_1.2 and the functional consequences of the two mutants reported here would be exerted in human neurons co-expressing K_Na_1.1 and K_Na_1.2.

In the present study, we did not investigate the cellular mechanisms that would account for the effects of the two mutations. First, we showed that both mutant subunits failed to generate significant current. This was expected for K_Na_1.2^L48Qfs43^ as the mutant subunit would not contain a pore domain—if not degraded. This was also not surprising for K_Na_1.2^K564*^ as the truncated part of the C-terminal domain includes the RCK2 domain which contains coordination motif for Na^+^ interaction (Thomson et al., 2015). Second, cells co-transfected with plasmids encoding K_Na_1.1 and either of the mutant K_Na_1.2 subunits in a homozygous state exhibited whole-cell currents that were similar to the currents measured in cells transfected with the K_Na_1.1 plasmid alone. This suggested either that the mutant subunits are rapidly degraded or lead to a non-functional heteromeric channel, or that the mutations prevented the assembly of K_Na_1.2 with K_Na_1.1. Indeed, the N-terminal domain of K_Na_1.1 plays a key role in heteromerization and channel trafficking (Chen et al., 2009). As channel formation (assembly, stabilization, trafficking) generally involves multiple inter-subunit association sites (Deutsch, 2002), it is also possible that the lack of the C-terminal part of mutant K_Na_1.2 subunits plays instrumental role.

In the K_Na_1.1/K_Na_1.2^WT^/K_Na_1.2^K564*^ mutant heterozygous configuration mimicking the patient situation, global current density was intermediate between that of cells expressing K_Na_1.1 alone and that of cells expressing both K_Na_1.1/K_Na_1.2 subunits. Whether the moderate alteration (~25% decrease) of K_Na_ current density observed with the p.K564* mutation would be sufficient to cause EIFMS remains to be firmly established. Indeed, *KCNT2* does not look that intolerant to heterozygous loss of function mutations, as can be inferred from databases of control individuals. On the one hand and although very unlikely, we cannot firmly exclude that the identification of the *de novo* p.K564* mutation in an EFMIS patient was coincidental by chance only. On the other hand, nonsense mutations in other epilepsy genes (e.g., *DEPDC5*) have also been detected in control individuals, and pLI scores should be interpreted with caution (Fuller et al., 2019). Also, different nonsense mutations in a given gene might be differently subjected to nonsense-mediated mRNA decay (NMD) which in turn sustains compensatory effects (El-Brolosy et al., 2019). Moreover, the actual impact of a deleterious ion channel mutation might be better seen in the genetic context of variants in other ion channels (Klassen et al., 2011). Interestingly, while the typical mutations of *KCNT1* leading to EIFMS are gain of function and *KCNT1* would be even more tolerant than *KCNT2* to loss-of-function variants (pLI score at 0.01 at the ExAC database, http://exac.broadinstitute.org/), a Phe932Ile loss of function variant in *KCNT1* was reported in a patient with severe epilepsy, delayed myelination and leukoencephalopathy (Vanderver et al., 2014; Evely et al., 2017), further indicating that decreased activity of K_Na_ channels can indeed be associated with severe neurological manifestations including epilepsy. The other mutation found here in *KCNT2*, p.L48Qfs43, had more dramatic effects than p.K564*: in the K_Na_1.1/K_Na_1.2^WT^/K_Na_1.2^ L48Qfs43^ configuration, current global density was more severely affected than with p.K564* and was similar to that of K_Na_1.1 alone, consistent with a dominant-negative effect of p.L48Qfs43. Overall this indicates how important these channels are to control neuronal excitability at early developmental stages. That similar phenotypes were observed in patient B carrying the p.L48Qfs43 mutation (~55% decrease K_Na_ current density) or in a patient carrying the gain of function p.R190P mutation (Ambrosino et al., 2018) suggests that K_Na_ channels efficiency should be tightly regulated during brain development and that any alteration, whatever its direction, would deeply impact on cortical networks activities.

There is now evidence that gain or loss of function mutations of a given ion channel may both lead to epileptic encephalopathies—although differences in phenotypes may exist (see above). This has been well documented for Kv7.2 *de novo* mutations (Miceli et al., 2013, 2015; Orhan et al., 2014; Abidi et al., 2015; Devaux et al., 2016; Mulkey et al., 2017). We now show that this is also the case for K_Na_1.2 mutations. This may have practical implications as drugs inhibiting/reducing K_Na_1.1 channel activity such as quinidine have been used to improve the EEG and background activity in a subset of the patients. Different hypotheses have been proposed to explain how an increase or a decrease in the function of a given ion channel may have similar consequences on network activity. Notably different sensitivity of a mutant channel in pyramidal cells and in interneurons has been suggested, creating an imbalance between excitation and inhibition or favoring neuronal synchronization (Miceli et al., 2015; Niday and Tzingounis, 2018). The development of animal models carrying loss and gain of function mutations is needed to solve this apparent paradox. This is particularly important for the K_Na_1.2 subunit, whose exact role in neuronal activity remains to be addressed.

## Data Availability Statement

The raw data of WES were deposited at the Sequence Read Archive (SRA) public database at NCBI (https://www.ncbi.nlm.nih.gov/sra; accession numbers: PRJNA592898; release date 2020-01-31, and PRJNA593942, release date 2019-12-08). The KCNT2 variants were deposited at the ClinVar public database at NCBI https://www.ncbi.nlm.nih.gov/clinvar/; c.1690A>T: accession number VCV000695093.1; c.143-144 delTA: accession number VCV000695094.1).

## Ethics Statement

The studies involving human participants were reviewed and approved by Ethics Committee of Hunan Provincial Maternal and Child Health Care Hospital. Written informed consent to participate in this study was provided by the participants’ legal guardian/next of kin. Written informed consent was obtained from the individual(s), and minor(s)’ legal guardian/next of kin, for the publication of any potentially identifiable images or data included in this article.

## Author Contributions

XM designed and performed clinical investigations and genetic analyzes. NB designed and performed cell biology experiments (expression constructs, cell cultures and transfections). HB participated in the electrophysiological experiments. QG, ZJ, and HW collected clinical data. HX and LS performed data analysis. PS coordinated and participated in the design of the overall study. LA designed, performed, analyzed and coordinated the electrophysiological experiments and wrote the article with the help of XM, NB, and PS.

## Conflict of Interest

The authors declare that the research was conducted in the absence of any commercial or financial relationships that could be construed as a potential conflict of interest.
